# Cervical angiofibroma of soft tissue: A rare case report with literature review

**DOI:** 10.1097/MD.0000000000040200

**Published:** 2024-10-18

**Authors:** Yaoqi Shi, Yuhong Xu, Minhua Li, Weiping Zheng, Jiangjing Shan

**Affiliations:** a Department of Gynecology, Shaoxing People’s Hospital, Shaoxing, China; b Department of Pathology, Shaoxing People’s Hospital, Shaoxing, China.

**Keywords:** angiofibroma of soft tissue, cervix, diagnosis, NCOA2 rearrangement

## Abstract

**Rationale:**

Angiofibroma of soft tissue (AFST) is a rare benign fibrous tumor recently included in the 2020 WHO classification of soft tissue and bone tumors. Currently, there are limited reports on AFST, and pathologists lack sufficient understanding of its clinical and pathological characteristics. There is scarce literature available on AFST in the cervical region.

**Patient concerns:**

We presented a case of a 49-year-old woman who was admitted to our hospital for emergency treatment due to vaginal bleeding and fatigue. Ultrasound revealed a 63 × 27 mm hypoechoic mass extending from the cervical opening to the external opening, with a hemoglobin level of 58.0 g/L on blood routine.

**Diagnoses:**

The tissue exhibited a pale-yellow mucinous appearance with distinct tissue boundaries and a fibrous capsule under a microscope. HE staining revealed spindle-shaped fibroblast-like cells with a consistent morphology, thin-walled branching small blood vessels, and dilated blood vessels. Regions with abundant cells and areas with sparse cells were alternately distributed and migrated gradually. Immunohistochemical analysis indicated positive expression of P53, desmin, progesterone receptor, estrogen receptor, epithelial membrane antigen, vimentin, CD68, CD163 in the tumor, but negative expression of P16, S-100, smooth muscle actin, CD117, CD10, STAT-6. CD34 was negative in the tumor cells but positive in the vascular endothelium. The Ki-67 index value was 5%. Pathological examination confirmed a soft tissue angiofibroma of the cervix.

**Interventions:**

Emergency hysteroscopic surgery was performed following infusion of 3 units of packed red blood cells. A local excision of the cervical mass was performed.

**Outcomes:**

A 1-month follow-up ultrasound showed no abnormal mass in the cervical canal, and there have been no signs of recurrence to date.

**Lessons:**

Cervical angiofibroma of soft tissue is a rare tumor with a benign clinical manifestation, minimal local recurrence, and no significant metastatic potential. Treatment primarily involves local resection with a focus on achieving negative surgical margins. By presenting this case, we aim to enhance the diagnostic and differential diagnostic capabilities of pathologists in identifying uterine tumors and preventing misdiagnosis.

## 1. Introduction

Angiofibroma of the soft tissue (AFST) is a benign fibroblastic/myofibroblastic tumor that typically occurs in the lower limbs. To date, there have been few reports of AFST in the cervical area. Histologically, AFST is characterized by a rich vascular supply and mucinous matrix degeneration, which can result in misdiagnosis as other soft tissue tumors with similar features, such as vascular tumors and mucinous soft tissue tumors. Our hospital has recently treated a case of AFST in the cervical region. Immunohistochemistry and fluorescence in situ hybridization (FISH) were performed on the pathological tissues. A thorough literature review was conducted to enhance the clinical pathologists’ understanding of this tumor, with the aim of preventing misdiagnosis and overtreatment.

## 2. Case presentation

**Figure 1.**
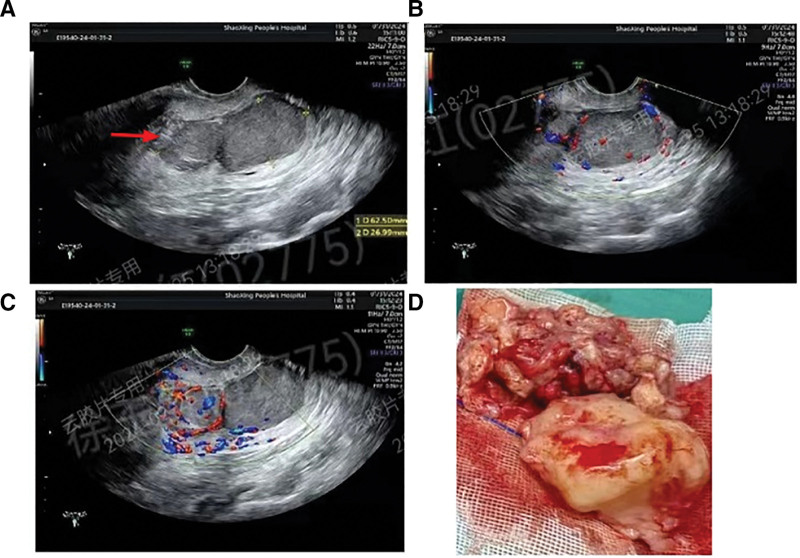
(A–C) Ultrasound images of the tumor. (D) The gross of the excised tumor.

A 49-year-old married woman was admitted to our hospital on January 30, 2024, with 14 days of vaginal bleeding and 1 week of fatigue. Her previous menstrual cycle was regular, with a 30-day cycle and a 4-day menstrual period, characterized by moderate flow and absence of menstrual pain. She denied any previous history of chronic diseases and had no personal or family history. An emergency routine blood examination revealed a hemoglobin level of 58.0 g/L. A transvaginal ultrasound of the uterine adnexa showed a 63 × 27 mm hypoechoic mass extending from the inner cervical opening to the outer opening, appearing to consist of 2 stacked pieces with blood flow signals, indicating a possible cervical fibroid (Fig. 1A–C). Endometrial thickness was measured at 10mm. Tumor markers such as CA125, CA199, HCG, and SCC were within the normal ranges. The patient received an emergency infusion of 3 units of type A RH positive suspended red blood cells and was admitted to the gynecology department of our hospital. Subsequent blood tests on January 31, 2024, showed a hemoglobin level of 75.0 g/L. Emergency surgery was performed the same day. During the surgery, a fibroid obstruction was identified at the cervical opening, with fibroid-like masses measuring 4.0 × 3.0 cm and 3 × 2.5 cm observed in the cervical canal. The pedicle was broad and extended locally into the muscular layer, with no significant thickening of the endometrium noted. Hysteroscopic resection of the uterine lesion and diagnostic curettage were performed. Due to the tumor’s location, hysteroscopy was utilized to electrically excise the tumor until complete removal was achieved. The surgical specimen was fragmented (Fig. 1D). The tissue presented a pale-yellow mucinous appearance.

**Figure 2.**
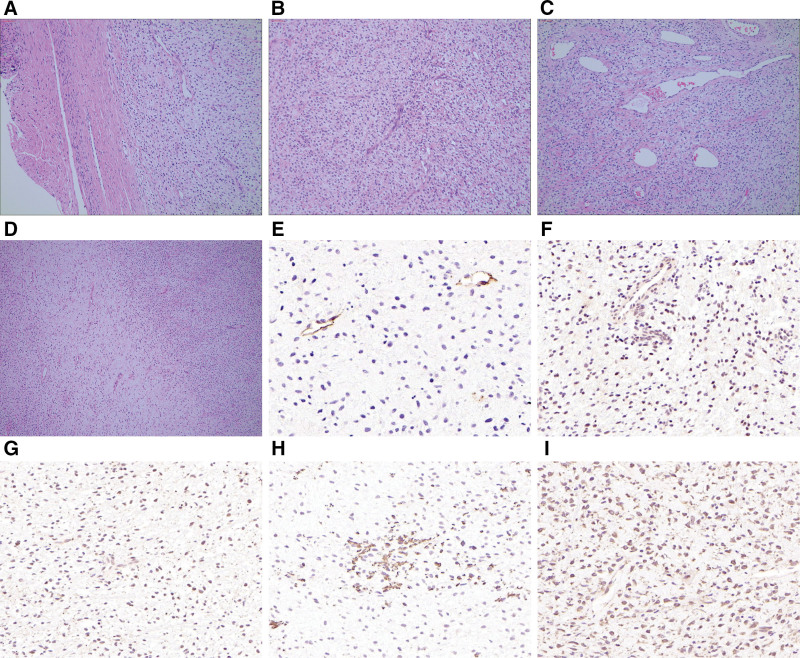
HE staining and immunohistochemical results of the tumor. (A) The tumor exhibited clear boundaries and a fibrous capsule (HE × 100). (B) The tumor was composed of relatively consistent, mild fibroblast-like spindle cells and thin-walled branching blood vessels, distributed in mucinous and collagen-like stroma (HE × 100). (C) Moderate to large circular, irregular or dilated blood vessels were observed surrounding the tumor (HE × 100). (D) The tumor displayed an alternating pattern of cellular-rich and cellular-loose areas, gradually transitioning (HE × 40). (E) CD34 was negatively expressed on the surface of tumor cells, clearly outlining the vascular structure (×200). (F) The expression of epithelial membrane antigen was positive (×200). (G) The expression of CD68 was positive (×200). (H) The expression of CD163 was positive (×200). (I) The expression of ER was positive (×200).

Under a microscope, the tissue exhibited clear boundaries and a fibrous capsule (Fig. 2A). HE staining revealed spindle-shaped fibroblast-like cells with relatively consistent morphology, thin-walled branching small blood vessels, and dilated blood vessels (Fig. 2B and C). Areas with abundant and loose cells were distributed alternately and migrated gradually (Fig. 2D). Immunohistochemical results showed that the tumor tested positive for P53, desmin, but negative for P16, S-100, smooth muscle actin (SMA), CD117 and CD10. CD34 was negatively expressed in the tumor cells and positively expressed in the vascular endothelium (Fig. 2E). The Ki-67 index value was 5%. We subsequently tested the epithelial membrane antigen (EMA) (Fig. 2F), vimentin, CD68 (Fig. 2G), and CD163 (Fig. 2H) in tumor tissues, all of which showed positive expression. Further immunohistochemical analysis at the Affiliated Obstetrics and Gynecology Hospital of Zhejiang University revealed negativity for anaplastic lymphoma kinase, CK, CK7, STAT-6, Caldesmon, and P-Trk, with positive staining for Rb, progesterone receptor (PR), estrogen receptor (ER, Fig. 2I), and CyclinD1 (<10%). A diagnosis of cervical fibroblastoma/myofibroblastic tumor, consistent with AFST, was confirmed. Fluorescence in situ hybridization showed no NCOA2 gene splitting.

The patient’s vaginal bleeding significantly decreased post-surgery and ceased after 7 days. A follow-up 1-month later showed normal hemoglobin levels in the blood routine examination. Ultrasound examination showed no abnormal mass in the cervical canal and there have been no signs of recurrence thus far.

## 3. Discussion

In 2012, Mariño-Enríquez and Fletcher first reported and officially named soft tissue angiofibroma (AFST) due to its histological features of significant fibroblast proliferation and formation of a distinct interstitial vascular network.^[1]^ AFST is more common in women and usually occurs in the lower limbs, often involving adjacent large joints. It is also occasionally seen in the back, abdominal wall, pelvic cavity, and breasts.^[2]^ Overall, most AFSTs have clear boundaries, some may have fibrous capsules, and a few may infiltrate adjacent soft tissues. The cut surface is gray-white, gray-red, and gray-yellow, with a soft texture appearing as mucinous, gelatinous, or edematous. Those that are hard and tough have a rubber-like appearance and are elastic. Histologically, the typical AFST is mainly composed of relatively consistent short spindle-shaped or spindle-shaped fibroblast-like cells and abundant thin-walled branching small blood vessels, distributed in a mucoid, fibromucoid, or collagen matrix. The tumor cells had a mild morphology, no obvious atypia, few cytoplasms, and oval or spindle-shaped nuclei. The chromatin of the nucleus was delicate, with occasional small nucleli. A few cases may present with degeneration of the tumor cell nuclei and multinucleated giant cells. Vascular components within the tumor are relatively complex. In addition to abundant thin-walled branching small blood vessels, most cases are accompanied by a relatively small number of medium to large circular, irregular, or dilated blood vessels. The thickness of the blood vessel wall varies, and sometimes antler-like or branching blood vessels can be observed, forming a vascular exodermis-like structure. The vascular wall may be accompanied by glassy degeneration or cellulose-like necrosis. The tissue section of this case presented a pale-yellow mucinous appearance with clear tissue boundaries and a fibrous capsule under the microscope. HE staining revealed spindle-shaped fibroblast-like cells with a consistent morphology, thin-walled branching small blood vessels, and dilated blood vessels. Regions with dense and sparse cells were distributed alternately and migrated gradually, consistent with the histological description of AFST.

We conducted a literature review of AFST to summarize its clinical and pathological characteristics, as presented in Tables 1 and 2. Analysis of 131 reported cases revealed that AFST lacked specific immunohistochemical markers. Overall, the expression levels of CD68, CD163, ER, and vimentin were relatively high, whereas EMA and desmin also demonstrated positive expression. In contrast, SMA and S-100 showed predominantly negative expression, with a 100% negativity rate for STAT-6. CD34 was mostly negative in tumor cell membranes but positive in vascular endothelium. CD34 can delineate the contours and distribution of the blood vessels. The Ki-67 positivity index was ≤ 10%. According to Yamada et al,^[11]^ ER and CD163 positivity can aid in AFST diagnosis, but they lack specificity. Immunohistochemical analysis of this case indicated positive results for desmin, EMA, ER, CD68, and CD163, whereas SMA, S-100, and STAT-6 were negative. The Ki-67 positivity index was <10%. These immunohistochemical findings were generally consistent with those reported in previous studies related to AFST. Recent molecular genetic studies have suggested that AFST exhibits specific chromosomal translocations, with t (5; 8) (p15; q13) translocation leading to the AHRR-NCOA2 fusion gene. AHRR is a recognized tumor suppressor that regulates the activity of AHR, whereas NCOA2 acts as a transcriptional co-activator of nuclear hormone receptors. The function of the AHRR-NCOA2 chimeric protein has yet to be elucidated. However, activation of AHR signaling has been hypothesized to play a crucial role in the tumor transformation of AFST because of the preserved NCOA2 transcriptional activation domain.^[24]^ Arbajian et al^[4]^ discovered that a small proportion of AFST cases exhibit the GTF2I-NCOA2 fusion subtype. Panagopoulos et al^[12]^ proposed that AFST can also involve t (4; 5) (q24; q31) translocation, resulting in the formation of P4HA2-TBCK and TBCK-P4HA2 fusion genes. Additionally, t (5; 8; 17) (p15; q13; q21) translocation leads to the formation of the AHRR-NCOA2, EVT4-AHRR, and NCOA2-EVT4 fusion genes. According to Table 1, NCOA2 gene rearrangement is not always present in AFST; therefore, a negative NCOA2 result does not exclude the diagnosis of AFST. This is because NCOA2-positive cells may be rare and are only detected in a limited number of tumor cells. Chromogenic in situ hybridization (CISH) is a valuable tool for sensitive analysis of tumor cells and is more effective than reverse transcription polymerase chain reaction (RT-PCR) and FISH. Furthermore, since NCOA2 gene fusion can also occur in other malignancies, such as leukemia and sarcomas, NCOA2 is not a specific genetic marker for AFST.^[25,26]^

**Table 1:** Clinical features and molecular genetic analysis of reported cases related to angiofibroma of soft tissue.

NO	First author	Year	Sex (number)	Age (yr)	Site	Clinical treatment	Cytogenetic findings	Follow-up (mo)
1	Mariño-Enríque A^[1]^	2012	F (25) M (12)	6 to 86	Lower extremity (23), upper extremity (5), trunk (9)	SE (29), WE (6), amputation (1), N/A (1)	A balanced t(5;8)(p15;q12) translocation (4/6),3-way t(5;8;8)(p15;q13;p11) translocation (1/6)	NED (×23, 6–122 m), recurrence (4), N/A (9)
2	Schoolmeester JK^[3]^	2012	F (1)	54	Knee	SE	AHRR-NCOA2 fusion	N/A
3	Arbajian E^[4]^	2013	F (1)	41	Thigh	N/A	GTF2I/NCOA2 fusion	N/A
4	Edgar MA^[5]^	2013	F (1)	62	Iliac crest	Radical excision	NCOA2 gene rearrangement	NED (9 m)
			M (1)	67	Chest wall	SE	N/A	N/A
5	Zhao M^[6]^	2013	M (2)	57	Thigh	SE	N/A	NED (12 m)
				54	Posterior neck	SE		N/A
6	Song JS^[7]^	2014	F (1)	51	Thigh	SE	N/A	NED (unclear)
7	Fukuda Y^[8]^	2014	F (1)	73	Thigh	SE	NCOA2 gene rearrangement	NED (6 m)
8	Sugita S^[9]^	2014	F (2) M (2)	27 to 70	Thigh (2), upper arm (1), inguinal region (1)	SE (3), WE (1)	NCOA2 split (4/4)	NED (3–60 m)
9	Lee JJ^[10]^	2014	M (1)	37	Foot	SE	AHRR-NCOA2 fusion	NED (24 m)
10	Yamada Y^[11]^	2016	F (6) M (7)	28 to 70	Lower extremity (11), trunk (2)	N/A	NCOA2 gene rearrangement (9/13), negative (2/13), fail (2/13)	NED (×2, 4 m, 18 m), recurrence (×1, 96 m, N/A (×10)
11	Panagopoulos I^[12]^	2016	M (1)	45	Inguinal region	SE	P4HA2-TBCK, TBCK-P4HA2, AHRR-NCOA2, ETV4-AHRR, and NCOA2-ETV4 fusion	N/A
12	Jeong JW^[13]^	2017	F (1)	50	Cheek	SE	NCOA2 gene rearrangement	N/A
13	Hashino Y^[14]^	2017	F (1)	23	Knee	SE	N/A	NED (9 m)
14	Bekers EM^[15]^	2017	F (6) M (8)	7 to 67	Lower extremity (12), upper back (1), flank (1)	SE	AHRR-NCOA2 fusion(9/14),GAB1-ABL1 fusion(1/14)	NED (×10, 3–48 m), N/A (×4)
15	Ma HJ^[16]^	2018	F (1)	36	Temporal lobe	SE	N/A	NED (20 m)
			F (1)	59	Popliteal fossa			NED (16 m)
			M (1)	62	Lower extremity			N/A
16	Xu XL^[17]^	2018	F (9) M (15)	8 to 68	Upper extremity(3), lower extremity (11), trunk (7), temporal region(1), Retroperitoneum(1), liver(1)	SE(20), WE(2), Left kidney and retroperitoneal tumor resection(1), Partial liver resection(1)	NCOA2 gene rearrangement(3/4)	NED (×17, 3–69 m)
17	Ali Z^[18]^	2019	F (1)	55	Foot	SE	N/A	NED (unclear)
18	Yang DP^[19]^	2021	F (1)	52	Liver	WE	N/A	NED (to 2021.5.1)
19	Wang CM^[20]^	2022	F (5) M (3)	29 to 69	Lower extremity (8)	SE (5), WE (3)	NCOA2 gene rearrangement(7/8)	NED (5–73 m)
20	Nakayama S^[21]^	2022	F (1)	55	Knee	SE	AHRR-NCOA2 fusion	NED (12 m)
21	Han RM^[22]^	2023	F (1)	33	Cervicothoracic junction	SE	N/A	NED (14 m)
22	Yamashita K^[23]^	2023	F (7) M (5)	32-89	Lower extremity (11), upper extremity(1)	SE (3), WE (7), biopsy (2)	AHRR::NCOA3 fusion(2/12),AHRR::NCOA2 fusion(10/12)	NED (×7, 4–105 m), N/A (3), AWD(2)

Abbreviations: AWD = alive with disease, F = female, M = male, m = months, N/A = unavailable, NED = no evidence of disease, SE = simple excision, WE = wide excision.

Note: Lower extremity includes foot, ankle, popliteal fossa, knee, thigh, and lower leg. Upper extremity includes the wrist, arm and forearm. Trunk includes the back, pelvic cavity, breast, inguinal area, iliac crest, pectoralis muscle, and abdominal wall.

**Table 2:** Immunohistochemical results of reported cases related to angiofibroma of soft tissue.

No.	First author	Number of cases	EMA(+)	Desmin(+)	CD34 (+)	Vimentin (+)	SMA (+)	S-100(+)	STAT-6(+)	CD68(+)	CD163(+)	ER (+)	Ki-67/MIB-1 (%)
1	Mariño-Enríquez A^[1]^	37	16/36	4/35	5/36	N/A	5/35	0/36	N/A	N/A	N/A	N/A	N/A
2	Schoolmeester JK^[3]^	1	N/A	N/A	N/A	N/A	N/A	N/A	N/A	N/A	N/A	N/A	N/A
3	Arbajian E^[4]^	1	N/A	N/A	N/A	N/A	N/A	N/A	N/A	N/A	N/A	N/A	N/A
4	Edgar MA^[5]^	2	N/A	1/1	0/1	N/A	0/1	0/1	N/A	N/A	N/A	N/A	N/A
5	Zhao M^[6]^	2	0/2	2/2	Tumor cells CD34(−), endothelium CD34(+)	2/2	0/2	0/2	N/A	N/A	N/A	N/A	<1%
6	Song JS^[7]^	1	1	0	Tumor cells CD34(−), endothelium CD34(+)	N/A	0	0	N/A	N/A	N/A	N/A	<1%
7	Fukuda Y^[8]^	1	1	N/A	Endothelium CD34(+)	1	N/A	N/A	N/A	1	1	N/A	N/A
8	Sugita S^[9]^	4	3/4	4/4	0/4	4/4	1/4	2/4	0/4	N/A	N/A	3/4	N/A
9	Lee JJ^[10]^	1	0	0	N/A	1	0	0	N/A	N/A	N/A	N/A	<1%
10	Yamada Y^[11]^	13	4/13	6/13	0/13	N/A	0/12	N/A	0/13	7/13	13/13	13/13	1% to 6%
11	Panagopoulos I^[12]^	1	N/A	N/A	Endothelium CD34(+)	N/A	N/A	N/A	N/A	N/A	N/A	N/A	<5%
12	Jeong JW^[13]^	1	N/A	1	0	N/A	1	0	N/A	1	N/A	N/A	3%
13	Hashino Y^[14]^	1	0	1	0	1	0	0	N/A	1	1	1	N/A
14	Bekers EM^[15]^	14	7/9	2/6	1/13	N/A	2/11	0/11	0/5	N/A	N/A	N/A	N/A
15	Ma HJ^[16]^	3	0/3	3/3	Tumor cells CD34(−), endothelium CD34(+)	N/A	3/3	N/A	N/A	N/A	N/A	N/A	<1%
16	Xu XL^[17]^	24	2/4	1/10	Tumor cells CD34(+) 1/7	24/24	1/18	0/24	N/A	N/A	N/A	1/2	<5%
17	Ali Z^[18]^	1	1	N/A	1	N/A	0	0	N/A	N/A	N/A	N/A	N/A
18	Yang DP^[19]^	1	N/A	N/A	1	N/A	1	N/A	N/A	N/A	N/A	N/A	N/A
19	Wang CM^[20]^	8	3/8	4/8	Tumor cells CD34(−), endothelium CD34(+)	N/A	0/8 (tumor cells)	0/8	0/8	8/8	N/A	N/A	<1%
20	Nakayama S^[21]^	1	0	0	0	N/A	0	0	N/A	1	1	1	1% to 2%
21	Han RM^[22]^	1	1	0	Endothelium CD34(+)	N/A	N/A	0	0	N/A	1	N/A	Hot spot10%
22	Yamashita K^[23]^	12	5/12	9/12	0/12	N/A	1/12	0/12	0/12	12/12	12/12	10/12	1% to 7%
	Total	131	44/98	38/100	/	33/33	14/113	2/105	0/43	31/37	29/29	29/33	/

Abbreviations: EMA = epithelial membrane antigen, ER = estrogen receptor, N/A = unavailable, SMA = smooth muscle actin.

Note: 16/36 = 36 cases were tested, of which 16 cases were positive.

AFST must be distinguished from other soft tissue tumors that are rich in vascular and/or fibromyxoid matrix, such as low-grade myxofibrosarcoma (LGMFS), low-grade fibromyxoid sarcoma (LGFMS), myxoid liposarcoma (MLPS), solitary fibrous tumor (SFT), and cellular angiofibroma (CAF). Wang et al^[20]^ provided a detailed explanation of the differential diagnosis between these tumors and AFST, which will not be further elaborated in this article. SFT diffusely expresses CD34, STAT-6, CD99, and bcl-2, and harbors a NAB2-STAT-6 fusion gene. Immunohistochemistry of these tumor cells was negative for STAT-6, CD34, SMA, and S-100. Based on HE staining, the aforementioned types of tumors are currently not considered. In the cervical region, AFST needs to be differentiated from other rare mesenchymal tumors in the cervical and vaginal regions, such as superficial cervical fibroblastoma (SCVM), angiomyofibroblastoma (AMF), CAF, invasive angiomyxoma (AAM), and breast type myofibroblastoma (MTMF). SCVM comprises a group of benign mesenchymal tumors that typically arise in the submucosal matrix of the vagina and cervix in women. They present as polypoid or nodular lesions visible to the naked eye with glossy mucus on the cut surface. SCVM has a well-defined boundary with surrounding tissues but lacks a capsule, and a Grenz zone can be observed between the tumor and epithelium. The tumor tissue exhibits sparse tumor cells and a mucoedema-like stroma in the superficial area, whereas the central area shows densely arranged spindle cells and astrocytes with oval or wavy nuclei. The chromatin distribution is uniform, and mitosis is absent. Positive staining for desmin, vimentin, ER, PR, and CD34 is observed, with some cases showing SMA positivity.^[27]^ AMF is more commonly found in the external genitalia and inguinal region, and less frequently in the vagina. Under light microscopy, polynuclear cells are prominent, with an uneven distribution of tumor cells and alternating high and low cell areas surrounding blood vessels. The vessel wall appears edematous and mucinous with glassy changes. In addition to desmin, tumor cells may also express vimentin, with a small subset showing positivity for α-SMA and muscle specific actin (MSA).^[28]^ CAF is a rare benign tumor primarily located in the superficial soft tissues, more commonly affecting the female external genitalia, vagina, and male inguinal scrotum. Histologically, it is characterized by numerous medium-sized thick-walled blood vessels, clusters of adipocytes, fine collagen bundles, and active mitotic activity. Tumor cells typically do not express desmin or S-100.^[29]^ AAM is classified as a low-grade malignant tumor that predominantly arises in the deep soft tissues of the pelvic and perineal regions in young to middle-aged females. It features a large tumor size, indistinct boundaries, invasive growth, and high local recurrence rate. Histologically, the tumor is characterized by sparse cells, mucinous stroma, unevenly sized and distributed blood vessels, and infiltration of inflammatory cells around vessels. Immunophenotyping may reveal various combinations of positivity for vimentin, desmin, SMA, MSA, CD34, ER, and PR.^[30]^ MTMF is a benign tumor commonly found in the inguinal, vulvar, perineum, scrotum, and para-testicular regions. Microscopically, it consists mainly of spindle-shaped myofibroblasts and singly arranged Z-shaped collagen bundles. When it occurs in the vagina, its morphology resembles that of SCVM, but positivity for CD34, CD10, and SMA is often observed.^[31]^

Local resection with negative margins is the primary treatment approach for AFST, along with surgical resection for local recurrence. The follow-up data in Table 1 indicates a low recurrence rate of AFST, with only 5 of the 90 follow-up patients experiencing recurrence. A recent study indicated that malignant melanoma can metastasize to genetically confirmed soft tissue angiofibroma. Tumor-to-tumor metastasis is a relatively rare phenomenon that should be taken seriously.^[32]^

## 4. Conclusions

In summary, AFST is a distinctive fibrovascular soft tissue tumor that is predominantly benign, with low rates of local recurrence. The prognosis is generally favorable. A typical AFST is characterized by spindle-shaped fibroblast-like cells with uniform morphology, embedded in a fibromucinous matrix abundant in delicate branching blood vessels. AFST lacks specific immunohistochemical markers. CD163, CD68, ER, vimentin and EMA are commonly expressed. Some AFSTs exhibit NCOA2 gene-related translocations, which can be differentiated from other tumors using FISH, RT-PCR, or CISH if necessary.

## Acknowledgments

The authors are grateful to the pathologists at the Pathology Department of Zhejiang University Affiliated Obstetrics and Gynecology Hospital for their assistance in the diagnosis of this case.

## Author contributions

**Funding acquisition:** Yuhong Xu, Jiangjing Shan.

**Investigation:** Minhua Li.

**Project administration:** Yuhong Xu.

**Writing – original draft:** Yaoqi Shi.

**Writing – review & editing:** Yuhong Xu, Weiping Zheng, Jiangjing Shan.
